# Investigating the interaction between neuropsychiatry features and daily activities on social function in patients with Parkinson's disease with mild cognitive impairment

**DOI:** 10.1192/bjo.2022.611

**Published:** 2022-11-25

**Authors:** Yi-Ru Chen, Chun-Hsiang Tan, Hui-Chen Su, Chung-Yao Chien, Pi-Shan Sung, Tien-Yu Lin, Tsung-Lin Lee, Rwei-Ling Yu

**Affiliations:** Institute of Behavioral Medicine, College of Medicine, National Cheng Kung University, Taiwan; Department of Neurology, Kaohsiung Medical University Hospital, Kaohsiung Medical University, Taiwan; and Graduate Institute of Clinical Medicine, College of Medicine, Kaohsiung Medical University, Taiwan;; Department of Neurology, National Cheng Kung University Hospital, College of Medicine, National Cheng Kung University, Taiwan; Department of Neurology, National Cheng Kung University Hospital, College of Medicine, National Cheng Kung University, Taiwan;

**Keywords:** Parkinson's disease, mild cognitive impairment, social functioning, assessment tool, influence variables

## Abstract

**Background:**

Social functioning is crucial for daily living and is an essential indicator of dementia in patients with Parkinson's disease. The pattern of social functioning in patients with Parkinson's disease without dementia (i.e. those who are cognitively intact or have mild cognitive impairment (PD-MCI)) and its determinants are unclear.

**Aims:**

In exploring the heterogeneity of social functioning among patients with Parkinson's disease-associated dementia, we determined the optimal cut-off score of the Parkinson's Disease Social Functioning Scale (PDSFS) for patients with PD-MCI, and the variables influencing patients’ social functioning.

**Method:**

A total of 302 participants underwent the Mini-Mental State Examination (MMSE) and PDSFS; 120 patients with Parkinson's disease completed the measurements (MMSE, Activities of Daily Living Scale and Neuropsychiatric Inventory). Group comparisons, receiver operating characteristic curves, Spearman correlation and multiple and hierarchical regression analyses were conducted.

**Results:**

The PD-MCI group scored the lowest on the PDSFS (*F* = 10.10, *P* < 0.001). The PDSFS cut-off score was 53 (area under the curve 0.700, sensitivity 0.800, specificity 0.534). The MMSE (*β* = 0.293, *P* = 0.002), Activities of Daily Living Scale (*β* = 0.189, *P* = 0.028) and Neuropsychiatric Inventory (*β* = −0.216, *P* = 0.005) scores predicted the PDSFS score. Further, there was an interaction effect between the Activities of Daily Living Scale and Neuropsychiatric Inventory scores on the PDSFS score (*β* = 0.305, *P* < 0.001).

**Conclusions:**

We determined a PDSFS cut-off score for detecting PD-MCI and found that patients with PD-MCI have social dysfunction. Future research should focus on the effects of neuropsychiatry symptoms and activities of daily living on social functioning, and tailor the intervention programme for patients with Parkinson's disease.

## Social functioning among patients with Parkinson's disease

Parkinson's disease is a common neurodegenerative disease characterised by motor symptoms, and is the fastest-growing cause of disability arising from neurological disorders. Many studies have explored non-motor symptoms;^1–6^ however, social functioning has been neglected.^7^ Social functioning is an individual's ability to live independently and interact with society.^8^ Social dysfunction can sometimes represent the first signs of several neuropsychiatric disorders, manifesting long before the full onset of the other symptoms.^9–12^ Research has shown that Parkinson's disease affects the performance of social role functioning, which is related to overall happiness and quality of life, including couple relationships, friendship roles and work roles.^13^ There is also evidence that some patients with Parkinson's disease might even experience a change in the quality of social interaction a few years before they start experiencing extrapyramidal motor symptoms.^10,14^

In addition to its impact on life, independent daily function is key to distinguishing patients with Parkinson's disease-associated dementia, mainly from patients with Parkinson's disease with mild cognitive impairment (PD-MCI).^15^ According to the Movement Disorder Society (MDS) diagnostic criteria for PD-MCI, patients may have slight difficulties in complex functional tasks, but cognitive impairment is insufficient to interfere with functional independence.^16^ Specifically, compared with patients with Parkinson's disease-associated dementia, social functioning in patients with PD-MCI should be intact. Recently, our team developed the Parkinson's Disease Social Functioning Scale (PDSFS) to measure social functioning in Parkinson's disease. The PDSFS can determine whether a patient has Parkinson's disease-associated dementia.

Moreover, our previous study found that some patients with Parkinson's disease and dementia may also have social dysfunction.^8^ Social functioning research is new in the Parkinson's disease population,^8,17,18^ but few studies have explored social functioning in PD-MCI. Notably, the study by Pirogovsky et al, which was not focused on social function, was the most similar to ours. They found that the PD-MCI group scored lower on medication and financial management ability than the healthy group. Cognitively intact patients with Parkinson's disease (PD-CI) did not show difficulties with this ability.^19^

## Relevant factors may influence social functioning

Sommerlad and Rapaport mentioned that few studies have directly targeted interventions for social functioning, and improvements in social functioning are often the result of other interventional programmes (e.g. cognitive stimulation therapy).^20^ There is limited research on factors that may affect social functioning for patients with Parkinson's disease, and it is mainly focused on the impact of severity or motor symptoms of the disease on social functioning.^13,18,21^

Most other studies exploring factors that affect social functioning (e.g. cognitive function, neuropsychiatric symptoms and activities of daily living) were on patients without Parkinson's disease. Cognitive impairments^9^ and neuropsychiatric symptoms may affect social functioning. Evidence shows that more severe depression or anxiety symptoms may worsen social functioning.^22^ Finally, some studies have pointed out that the more independent the activities of daily living, the better the social functioning.^20^

## Unmet need for the field of social functioning among patients with Parkinson's disease

Social functioning affects an individual's daily life and can be used to determine whether an individual has dementia. Whether patients with Parkinson's disease have social dysfunction before the onset of dementia and the comprehensive picture of social functioning among patients with PD-MCI require further exploration. Moreover, the factors that affect the social functioning of patients with Parkinson's disease should be investigated to develop further interventions.

## Aims

We aimed to extend our previous study^8^ to determine the cut-off point for PD-MCI on the PDSFS, and explore the social functioning picture among patients with PD-MCI. We also aimed to investigate the factors that may affect social functioning among patients with Parkinson's disease and determine their relationship, to better understand the key to social functioning.

## Method

### Participant

We recruited patients with Parkinson's disease in the out-patient setting of two medical centres (National Cheng Kung University Hospital and Kaohsiung Medical University Hospital) and older adults in the community. A total of 181 patients with Parkinson's disease-associated dementia and 121 healthy older adults were recruited for our primary aim. All of the patients with Parkinson's disease were referred by neurologists and were required to meet the UK Parkinson's Disease Society Brain Bank Clinical Diagnosis Criteria^23^ and be diagnosed with idiopathic Parkinson's disease. We excluded atypical features of Parkinsonism, compliance with Parkinson's disease-associated dementia diagnostic criteria,^24^ previous severe traumatic brain injury or brain surgery (e.g. deep brain stimulation), other systemic severe diseases (e.g. heart failure, severe liver cirrhosis, malignancy), comorbid serious mental illness (e.g. major depressive disorders, general anxiety disorder) and other neurological disorders (e.g. Alzheimer's disease, epilepsy), as well as patients who were unable to cooperate to complete the test.

Healthy older adults were recruited from the community. This group included older adults over the age of 50 years and excluded those with Mini-Mental State Examination (MMSE) scores <24 and other excluded conditions, as mentioned above for patients with Parkinson's disease.

We enrolled 120 patients with Parkinson's disease to elucidate our second aim. The inclusion and exclusion criteria were the same as those described above, except for the exclusion condition of compliance with Parkinson's disease-associated dementia diagnostic criteria. To gain a complete understanding of the factors that may affect the social functioning of Parkinson's disease, all patients with Parkinson's disease with various severities of cognitive impairment were included. If participants in the primary aim met the inclusion criteria of the secondary aim, with no data missing, they would be included in our secondary aim. The primary and secondary aim participants partially overlapped (*n* = 97).

The authors assert that all procedures contributing to this work comply with the ethical standards of Taiwan and institutional committees on human experimentation and the Helsinki Declaration of 1975, as revised in 2008. The Kaohsiung Medical University Hospital (Kaohsiung, approval number: KMUHIRB-G(II)-20160001) and National Cheng Kung University Hospital (Tainan, approval number: A-ER-107-425) approved procedures involving human participants. All participants (or a legally authorised representative) were asked to sign a written informed consent form after they had agreed to participate in our study. Data collection was undertaken in laboratories.

### Measurements

The MMSE^25^ was used to measure cognitive function. For the primary study aim, all patients with Parkinson's disease were divided into PD-CI and PD-MCI groups. The cut-off score for the MMSE established that patients with Parkinson's disease with MMSE scores >25 points were less likely to have MCI.^26^ The PDSFS^8^ evaluates the social functioning of patients with Parkinson's disease, and is a self-report questionnaire that takes approximately 10–15 min to complete. If the patient could not answer because of other factors, such as unconsciousness or illiteracy, the family member or primary caregiver could fill in the answer. There were 25 questions, with a total score of 68. The higher the score, the better the patient's social functioning. Additionally, there were three factors of the PDSFS: ‘family life, hobbies and self-care’; ‘interpersonal relationship and recreational leisure’ and ‘social bond’. Examples of questions for the three factors include: ‘Can you repair items in the house, or do you know how to repair items in the house?’ ‘appropriate reaction when interacting’ and ‘communication through electronic media’. Finally, part 3 of the MDS-sponsored revision of the Unified Parkinson's Disease Rating Scale^27^ was used to measure motor symptoms among patients with Parkinson's disease. The higher the score, the more severe the motor symptoms.

The second aim was to assess demographic and clinical information (e.g. age of onset, disease duration and Hoehn–Yahr stage). The PDSFS,^8^ MMSE,^25^ Activities of Daily Living Scale (ADLS),^28^ and Neuropsychiatric Inventory (NPI)^29^ were used to evaluate the social functioning, global cognitive functions, activities of daily living and neuropsychiatric symptoms of patients with Parkinson's disease.

### Statistical analyses

We used SPSS software (version 24 for Windows) for all statistical analyses. All hypothetical tests for inferential statistics set a significance level of 0.05. When the data are normally distributed, we use the parametric analysis, and when the data are non-normally distributed, we use the nonparametric analysis. For the primary aim, demographic variables and PDSFS score for healthy older adults, patients with PD-CI and patients with PD-MCI were tested with the *χ*^2^-test and Kruskal–Wallis test; the Mann–Whitney *U* test was used for clinical features. The Bonferroni correction was adjusted for multiple comparisons between the three groups described above. Analysis of covariance was used to compare the PDSFS between the three groups, after controlling for demographic variables (age and education). The receiver operating characteristic curve was used to determine the optimal cut-off score for PD-MCI on the PDSFS. For the secondary aim, Spearman's correlation was used for the demographic variables, clinical characteristics and test results related to the PDSFS. Multiple regression, hierarchical regression and simple slopes analysis were used to identify variables that could predict the PDSFS score and determine the relationship between variables.

## Results

### Demographic and clinical characteristics

The demographic and clinical characteristics of the two cohorts are summarised in Tables 1 and 2, respectively. Gender, age and education were the demographic variables that revealed significant differences among the three groups. The comparison of PDSFS scores revealed that healthy older adults scored the highest, followed by patients with PD-CI, and patients with PD-MCI scored the lowest (*P* < 0.001); the same pattern was also found for the PDSFS factor ‘social bond’. The healthy older adult and PD-CI groups scored significantly higher on the ‘family life, hobbies and self-care’ factor than the PD-MCI group. However, no significant difference among the three groups was found for the ‘interpersonal relationship and recreational leisure’ factor.

**Table 1:** Demographics and clinical characteristics in study groups for the primary aim

	Healthy adults	PD-CI	PD-MCI	*P*-value	*Post hoc* analysis
Sample size	121	126	55	—	—
Gender, male	32 (26.4%)	80 (63.5%)	36 (65.5%)	<0.001	Healthy adults < PD-CI = PD-MCI
Age, years	64.79 (7.38)	65.55 (8.90)	71.47 (9.63)	<0.001	Healthy adults = PD-CI < PD-MCI
Education, years	12.31 (3.91)	13.05 (3.78)	9.87 (5.48)	0.002	Healthy adults = PD-CI > PD-MCI
Age of onset, years	—	59.39 (10.39)	64.77 (10.51)	0.001	—
Disease duration, years	—	6.02 (5.98)	6.23 (4.48)	0.709	—
Hoehn–Yahr stage	—	1.97 (0.82)	2.19 (0.71)	0.059	—
LED	—	389.45 (390.65)	351.36 (367.28)	0.659	—
MDS-UPDRS part 3	—	33.00 (9.23)	34.77 (10.76)	0.303	—
PDSFS	52.63 (9.77)	49.45 (8.98)	43.36 (11.12)	<0.001	Healthy adults > PD-CI > PD-MCI
Family life, hobbies and self-care	23.45 (4.45)	22.56 (4.60)	20.00 (5.46)	<0.001	Healthy adults = PD-CI > PD-MCI
Interpersonal relationship and recreational leisure	20.85 (4.56)	21.18 (4.40)	19.27 (5.67)	0.130	—
Social bond	8.32 (3.38)	5.71 (3.11)	4.09 (2.85)	<0.001	Healthy adults > PD-CI > PD-MCI

Data are described as mean (s.d.) for continuous variables and as number (percentage) for categorical variables. PD-CI, cognitively intact with Parkinson's disease; PD-MCI, Parkinson's disease with mild cognitive impairment; LED, levodopa equivalent dosage; MDS-UPDRS, Movement Disorder Society-sponsored revision of the Unified Parkinson's Disease Rating Scale; PDSFS, Parkinson's Disease Social Functioning Scale; =, no significant difference; >, significantly more than; <, significantly fewer than.

**Table 2:** Demographics and clinical characteristics in the second cohort (total number of Parkinson's disease participants was 120)

	Mean	s.d.	Range
Gender, male	83 (69%)	—	—
Age	67.43	8.89	45–87
Education, years	11.90	4.81	0–23
Age of onset, years	61.21	9.79	38–84
Disease duration, years	6.06	4.31	1–20
Hoehn–Yahr stage	2.16	0.76	1–4
Mini-Mental State Examination	25.15	4.28	7–30
Activities of Daily Living Scale	92.88	18.34	0–100
Neuropsychiatric Inventory	5.87	11.43	0–79
PDSFS	46.96	11.88	11–65
Family life, hobbies and self-care	20.79	6.98	0–27
Interpersonal relationship and recreational leisure	21.27	5.01	4–27
Social bond	4.90	3.04	0–14

PDSFS, Parkinson's Disease Social Functioning Scale.

### Analysis of covariance on the PDSFS and its factors

After controlling for the demographic variables of gender, age and education, there was a significant difference in PDSFS score between the groups (*F* = 10.10, *P* < 0.001). After pairwise comparisons, the healthy older adults and patients with PD-CI scored similar, and higher than the patients with PD-MCI (Table 3). In the factor of ‘family life, hobbies and self-care’, a significant difference was found between groups after controlling for the demographic variables (*F* = 5.45, *P* < 0.005), and the pairwise comparisons showed the same pattern as the PDSFS. The ‘interpersonal relationship and recreational leisure’ factors found a significant difference between groups after controlling for the demographic variables (*F* = 3.35, *P* = 0.036). Pairwise comparisons showed that the PD-CI group scored significantly higher than the PD-MCI group. The healthy older adults showed no differences between the PD-CI and PD-MCI groups. In the last factor of ‘social bond’, a significant difference was found between groups after controlling for the demographic variables (*F* = 25.08, *P* < 0.001). Pairwise comparisons showed that healthy older adults scored the highest, followed by patients with PD-CI, and patients with PD-MCI scored the lowest.

**Table 3:** Analysis of covariance on the Parkinson's Disease Social Functioning Scale score and its factor (total number of participants was 302)

Dependent variable	Source of variation	Sum of squares	d.f.	Mean square	*F-*value	*P-*value	Pairwise comparisons
PDSFS	Gender	283.26	1	283.26	3.03	0.083	—
	Age	157.50	1	157.50	1.68	0.196	—
	Education	1.65	1	1.65	0.02	0.894	—
	Group	1890.50	2	945.25	10.10	<0.001	Healthy adults = PD-CI > PD-MCI
	Error	2770.16	296	93.62	—	—	—
Family life, hobbies and self-care	Gender	23.02	1	23.02	1.05	0.307	—
	Age	77.57	1	77.57	3.53	0.061	—
	Education	2.23	1	2.23	0.10	0.750	—
	Group	239.75	2	119.88	5.45	<0.005	Healthy adults = PD-CI > PD-MCI
	Error	6506.76	296	21.98	—	—	—
Interpersonal relationship and recreational leisure	Gender	33.60	1	33.60	1.52	0.219	—
	Age	2.60	1	2.60	0.12	0.732	—
	Education	28.33	1	28.33	1.28	0.259	—
	Group	148.54	2	74.27	3.35	0.036	PD-CI > PD-MCI
	Error	6558.17	296	22.16	—	—	—
Social bond	Gender	38.88	1	38.88	3.89	0.050	—
	Age	4.53	1	4.53	0.45	0.501	—
	Education	26.15	1	26.15	2.62	0.107	—
	Group	501.49	2	250.74	25.08	<0.001	Healthy adults > PD-CI > PD-MCI
	Error	2959.37	296	9.10	—	—	—

PDSFS, Parkinson's Disease Social Functioning Scale; PD-CI, cognitively intact with Parkinson's disease; PD-MCI, Parkinson's disease with mild cognitive impairment; =, no significant difference; >, significantly more than.

### Receiver operating characteristic of the PDSFS for PD-MCI

**Fig. 1:**
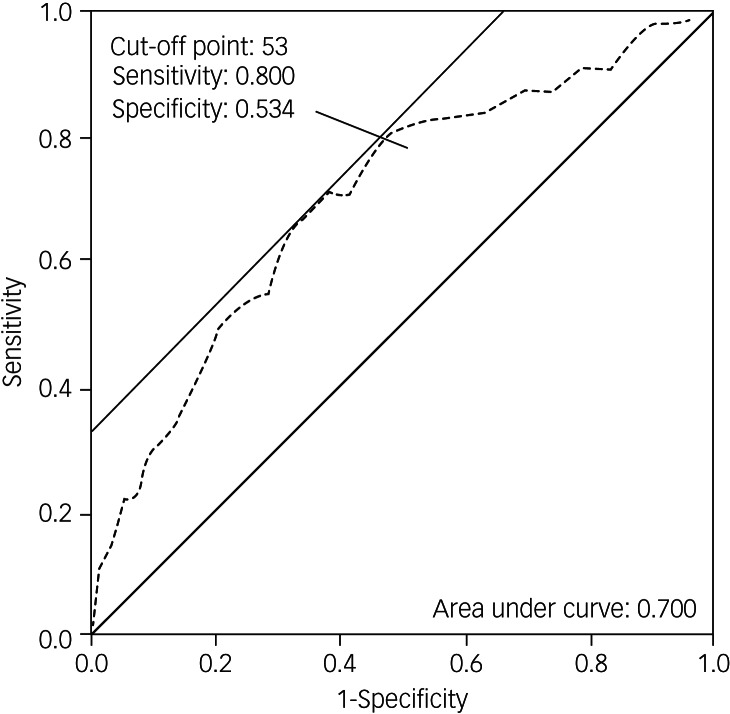
The receiver operating characteristic (ROC) curves of the Parkinson's Disease Social Functioning Scale (PDSFS) for patients with Parkinson's disease and mild cognitive impairment (PD-MCI). The ROC curves show the association between sensitivity and specificity on the PDSFS for PD-MCI. The grey dotted line represents the association between sensitivity and specificity on the PDSFS.

The PDSFS could distinguish PD-CI from PD-MCI (area under the curve, 0.700). The optimal cut-off score was 53 when the sensitivity was 0.800 and specificity was 0.534 (Fig. 1).

### Correlation between the PDSFS and relevant variables

The Spearman correlation between demographic variables, clinical characteristics, relevant factors (i.e. MMSE, ADLS and NPI), and the total PDSFS score among the 120 patients with Parkinson's disease are shown in Table 4. We found that in addition to gender and education, other variables were significantly correlated with the total PDSFS score.

**Table 4:** Correlation between the Parkinson's Disease Social Functioning Scale and the relevant variables in the second cohort (total number of Parkinson's disease participants was 120)

	Correlation coefficient	*P-*value
Gender	−0.169	0.064
Age	−0.398	<0.001
Education	0.058	0.529
Age of onset	−0.259	0.004
Disease duration	−0.206	0.024
Hoehn–Yahr stage	−0.374	<0.001
Mini-Mental State Examination	0.434	<0.001
Activities of Daily Living Scale	0.289	0.001
Neuropsychiatric Inventory	−0.329	<0.001

### Multiple regression analysis for predicting the PDSFS score

Multiple regression analysis was conducted to test whether age, age of onset, disease duration, Hoehn–Yahr stage, MMSE, ADLS or NPI could predict the total PDSFS score. Because of multicollinearity (variance inflation factor >10), the age of onset variable was deleted. The results showed that the MMSE (*β =* 0.293, *P* = 0.002), ADLS (*β =* 0.189, *P* = 0.028) and NPI (*β =* −0.216, *P* = 0.005) predicted the variance in PDSFS score (Table 5). Nevertheless, age, disease duration and Hoehn–Yahr stage were not significantly correlated with the PDSFS.

**Table 5:** Multiple regression for predicting the Parkinson's Disease Social Functioning Scale score using the second cohort data (total number of Parkinson's disease participants was 120)

	B	s.e.	*β*	*P*-value
Age	−0.138	0.116	−0.103	0.239
Disease duration	−0.270	0.204	−0.098	0.190
Hoehn–Yahr stage	−1.515	1.463	−0.096	0.303
MMSE	0.814	0.261	0.293	0.002
ADLS	0.122	0.055	0.189	0.028
NPI	−0.225	0.078	−0.216	0.005
*R*^2^	0.425			
Adjusted *R*^2^	0.395			
*F*-value	13.929			
*P*-value	<0.001			
d.f.	(6,113)			

MMSE, Mini-Mental State Examination; ADLS, Activities of Daily Living Scale; NPI, Neuropsychiatric Inventory.

### Interaction effect between variables on the PDSFS

**Fig. 2:**
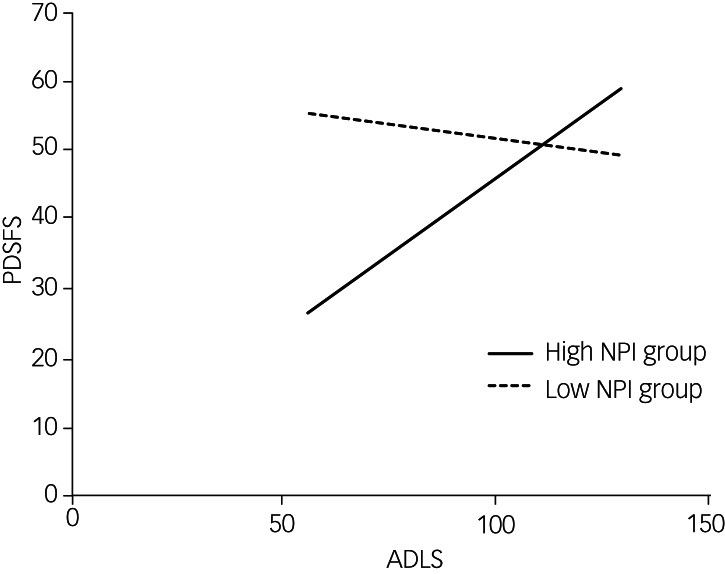
Interaction diagram of the Activities of Daily Living Scale (ADLS) and the Neuropsychiatric Inventory (NPI) on the Parkinson's Disease Social Functioning Scale (PDSFS). Patients were classified into low and high NPI groups (mean ± s.d.), and there was an interaction effect between the ADLS and NPI scores on the PDSFS score. The ADLS score range is presented as a mean.

Table 6 presents the results of the hierarchical regression analysis. First, the main effect of the ADLS and NPI could explain 28% of the PDSFS variation (*F* = 22.48, *P* < 0.001). After controlling for the main effect, the interaction between the ADLS and NPI increased the PDSFS variation by 7% (*F* = 13.29, *p* < 0.001). The interaction between the ADLS and NPI had a significant explanatory power for the PDSFS (*β* = 0.305, *P* < 0.001). An interaction diagram is shown in Figure 2. We used the mean and s.d. of the NPI to classify the patients into low (mean –1 s.d.) and high (mean +1 s.d.) NPI groups. The simple slope test showed that the ADLS had a significant explanatory power for PDSFS in the high NPI group (simple slope b = 0.439, *P* < 0.001) but not in the low NPI group (simple slope b = −0.087, *P* = 0.405).

**Table 6:** The interaction effect of each variable on the Parkinson's Disease Social Functioning Scale (total number of Parkinson's disease participants was 120)

	PDSFS					
	Δ*R^2^*	*P-*value^a^	*Β*	*P-*value^b^	Total *R^2^*	*P-*value^c^
Step 1	0.278	<0.001	—	—	0.352	<0.001
ADLS	—	—	0.387	<0.001		
NPI	—	—	−0.311	<0.001		
Step 2	0.074	<0.001	—	—		
ADLS	—	—	0.272	0.001		
NPI	—	—	−0.399	<0.001		
ADLS×NPI	—	—	0.305	<0.001		
Step 1	0.354	<0.001	—	—	0.372	<0.001
MMSE	—	—	0.450	<0.001		
ADLS	—	—	0.251	0.002		
Step 2	0.018	0.070	—	—		
MMSE	—	—	0.428	<0.001		
ADLS	—	—	0.146	0.141		
MMSE×ADLS	—	—	−0.178	0.070		
Step 1	0.347	<0.001	—	—	0.353	<0.001
MMSE	—	—	0.485	<0.001		
NPI	—	—	−0.223	0.005		
Step 2	0.006	0.289	—	—		
MMSE	—	—	0.467	<0.001		
NPI	—	—	−0.197	0.017		
MMSE×NPI	—	—	0.087	0.289		

PDSFS, Parkinson's Disease Social Functioning Scale; ADLS, Activities of Daily Living Scale; NPI, Neuropsychiatric Inventory; MMSE, Mini-Mental State Examination.

a. Significant value of Δ*R^2^*.

b. Significant value of *β*.

c. Significant value of total *R^2^*.

The main effect of the MMSE and ADLS could explain 35% of the PDSFS variation (*F* = 32.10, *P* < 0.001). After controlling for the main effect, the interaction between the MMSE and ADLS was not significant (*F* = 3.34, *P* = 0.07). Finally, the main effect of the MMSE and NPI explained 35% of the PDSFS variation (*F* = 31.02, *P* < 0.001). After controlling for the main effect, the interaction between the MMSE and NPI was not significant (*F* = 1.14, *P* = 0.289).

## Discussion

### Social functioning of patients with PD-MCI

After controlling for demographic variables, patients with PD-MCI scored the lowest in all domains of the PDSFS. Our findings confirmed that patients with Parkinson's disease without dementia might have declined social functioning.^8^ Additionally, our findings resonate with the PD-MCI diagnostic criteria of the MDS in that patients may have slight difficulties in complex functional tasks, but cognitive impairment is not enough to interfere with their functional independence.^16^

Regarding ‘family life, hobbies and self-care’, patients with PD-MCI performed worse than healthy older adults: for example, engaging in productive activities (e.g. doing housework or cooking), handling finances and managing transportation. Compared with the PD-CI group, the PD-MCI group performed worse in taking medicine, handling finances and managing transportation. These results are partly consistent with those of Pirogovsky et al, who found that patients with PD-MCI performed worse on medication and financial management than healthy older adults, and worse on medication management than patients with PD-CI.^19^ Our study further provides a more comprehensive and detailed picture of the social functioning of patients with PD-MCI; namely, that they may have more impaired complex social functions than healthy older adults and patients with PD-CI, particularly in areas of ‘family life, hobbies and self-care’ such as managing transportation.

Patients with PD-MCI performed worse than patients with PD-CI on ‘interpersonal relationship and recreational leisure’, especially in interpersonal communication, listening to family and friends, and interpersonal politeness. Regarding interpersonal communication, a recent study of semi-structured qualitative interviews found that patients with Parkinson's disease participated less in conversations;^14^ in the present study, this is particularly the case for patients with PD-MCI. For some patients, such changes in communication behaviour appear in the early stages of the disease.^14^ Changes in communication behaviour may be a result of patients having difficulty allowing themselves to be heard or seen in conversation. Another possibility is that patients with Parkinson's disease are less motivated to participate in social interaction.^14^

Regarding recreational leisure, studies have found that the types of activities that patients with Parkinson's disease engage in tend to be more solitary and sedentary, such as reading or watching television. They give up more physically demanding hobbies because of the disease, or favour more solitary activities because of the unpredictability of symptoms and embarrassment about symptoms.^13,30^ Therefore, we believe that patients with Parkinson's disease may experience subtle qualitative changes in interpersonal relationships and recreational leisure, and such changes are more pronounced among those with PD-MCI than PD-CI.

With regards to the ‘social bond’ factor, the PD-MCI group performed worse than the PD-CI group, especially in ‘communication by electronic media’ items. The PD-MCI group also performed worse than the healthy older adults group, especially in the items of ‘communication by electronic media’, ‘engaging in social activities’ and ‘inviting others to join the entertainment’. According to a recent literature review by Perepezko et al, friendships might change after diagnosis with Parkinson's disease.^13^ Some friendships are maintained, whereas others end.^13^ Through a smaller sample study, Rubenstein et al pointed out that patients with Parkinson's disease are less likely to initiate social outings with friends,^31^ which aligns with our results. The reasons for losing social connectedness might be that their disease prevents them from leaving the house or increases their desire to remain isolated to conceal symptoms.^13^

### Social functioning of patients with PD-CI

After controlling for demographic variables, we found that the overall social functioning of the PD-CI group was similar to that of healthy older adults. However, the PD-CI group scored significantly lower than healthy older adults in the ‘social bond’ domain of the PDSFS, especially in ‘engaging in social activities’, ‘inviting others to join entertainment’ and ‘community fair or gathering’. We found the ‘social bond’ deficit among patients with Parkinson's disease may not be related to the degree of cognitive function. Once an individual experiences the onset of Parkinson's disease, the ‘social bond’ domain might decline. Although the overall social functioning of the PD-CI group is not different from that of healthy older adults, the impairment of ‘social bond’ cannot be ignored. Social isolation and loneliness are harmful.^13,32^ In particular, the recent COVID-19 pandemic may exacerbate the problem of social connection,^33^ which requires more clinical attention.

Based on our findings, the optimal cut-off score of the PDSFS for PD-MCI was 53, and had acceptable discrimination. A possible reason for the low specificity is that the social function of PD-MCI and PD-CI was not yet severely impaired; therefore, the difference between the two was very subtle, and it may be difficult to distinguish between them. However, to the best of our knowledge, this is the first study to apply a social functioning scale to PD-MCI. This cut-off score was higher than the 39 points previously applied to patients with Parkinson's disease-associated dementia.^8^ These results indicated that if the total score of the PDSFS among patients with Parkinson's disease was between 40 and 53, they were likely to experience challenges with their social functioning when dealing with complex functional tasks. However, their functional independence was unaffected. We believe that the PDSFS can be used to clinically evaluate an individual's social functioning. The cut-off points for PD-MCI and Parkinson's disease-associated dementia^8^ can also be used as auxiliary information for diagnosing mild cognitive impairment or dementia in the Parkinson's disease population.

### Factors affecting social functioning in patients with Parkinson's disease

Our findings suggest that general cognitive function, activities of daily living and neuropsychiatric symptoms are significantly related to social functioning, whereas demographic and disease severity variables are not. Promoting social functioning in patients with Parkinson's disease can improve general cognitive function, facilitate activities of daily living and improve neuropsychiatric symptoms. A previous study on patients with dementia found that cognitive stimulation therapy^34^ benefits social interaction,^35^ and is the first management step suitable for patients with impaired social functioning.^20^ To the best of our knowledge, there is no direct evidence that promoting activities of daily living improves social functioning. However, patients with Parkinson's disease may engage in physical activities (i.e. dancing and boxing) that improve daily living,^36^ and have also been shown to be associated with better social functioning.^13,36,37^

We found that neuropsychiatric symptoms may influence patients’ social functioning. Neuropsychiatric symptoms were prevalent among patients with Parkinson's disease, and 57.5% had at least one neuropsychiatric symptom on the NPI. Studies have shown that social rehabilitation, which combines psychotherapy and cognitive rehabilitation among patients with chronic neuropsychiatric symptoms, can improve social functioning.^38^ Among patients with dementia, improving neuropsychiatric symptoms can also reduce the burden on caregivers, and thus improve their relationships with caregivers.^39^ Therefore, improving neuropsychiatric symptoms in patients with Parkinson's disease may also improve their social functioning.

We further found that neuropsychiatric symptoms may modulate the effect of activities of daily living on social functioning. On average, when the patient's NPI score was ≥6, their ADLS score had a significant positive explanatory power for PDSFS; however, this was not the case for the low NPI groups. Specifically, for patients with Parkinson's disease in the high NPI group, only when the ADLS score was poor, the individual could not maintain independent living or interact with society. A total of 28.3% of participants in our study had an NPI score of ≥6; the most common symptoms included sleep problems (82%), depression (76%), anxiety (68%) and apathy (62%). Medication can improve the above symptoms, but the side-effects caused by medication often cause treatment non-adherence among patients.^40^ Therefore, we suggest that improving social functioning by promoting activities of daily living may be a suitable non-pharmacological intervention method for this group of patients with high NPI score.

Based on our findings, we suggest that in the future, when formulating a programme to promote social functioning in patients with Parkinson's disease, cognitive training, activities of daily living or neuropsychiatric symptoms can be used as options. Moreover, assessing patients’ NPI score before planning the programme can help clinicians to tailor intervention plans.

### Limitations

First, we used the MDS level 1 recommendations to group the patients. We recommend that future studies refer to the level 2 approach of the MDS to subtype the PD-MCI group, to further explore social functioning in PD-MCI. Second, the number of participants in this study is small, and future studies need to recruit larger numbers of participants to further verify our findings. Third, the disease severity in our second cohort was relatively mild, and patients with Parkinson's disease at stage four are in the minority. We suggest that future studies cover a more comprehensive range of disease severity and increase the sample size to explore the possible effects on social functioning, as a reference for the future development of intervention programmes.

## Data Availability

The data that support the findings of this study are available from the corresponding author, R.-L.Y., upon reasonable request.
